# Endocarditis: Its Causes and Differential Diagnosis from the Pericarditis

**Published:** 1880-08

**Authors:** S. F. Carpenter

**Affiliations:** St. Joseph, Mo.


### ENDOCARDITIS: ITS CAUSES AND DIFFERENTIAL DIAGNOSIS FROM PERICARDITIS.

By S. F. CARPENTER, M.D., St. Joseph, Mo.

Few affections of the heart occur more frequently, or give rise to more symptoms than the inflammatory affection known as endocarditis. This affection, like nearly all inflammatory diseases, has both an idiopathic and traumatic origin; but outside of its idopathic origin it seldom occurs; as wounds and other injuries sufficient to cause this inflammation most generally results in speedy death. Therefore, in a large number of cases of endocarditis we must look to some other source and cause as its factor than traumatism.

The term endocarditis implies an inflammation of the endocardium or lining membrane of the heart. This inflammation may be acute or chronic, extensive or limited, as many cases have been found to be on post mortem examination. I have no doubt that many mild cases of this affection are overlooked when existing coincidently with rheumatism, Bright's disease or some other systemic affection.

We are not apt to suspect endocarditis, unless in a case of severe acute articular rheumatism, or protracted case of albuminuria; then, if found to exist, it is of such a character, and its symptoms and pathology so well established, that little difficulty will be had in the diagnosis.

I believe that all, or nearly all, authors agree that this inflammation is found to exist chiefly in the left or arterial side of the heart, but unless all post mortems reveal this fact our authors might, in many instances, be mistaken.

It is true that all ante-mortem physical examinations reveal the disease unmistakably in the left side of the heart, but this is not positive evidence that it does not exist in the right side also. When we consider that the left or arterial side of the heart lies almost in front of the right side, and owing to the walls of the left side being much thicker than the walls of the right, we can readily understand why it is that the sounds of the left, both normal and abnormal, can be heard more audibly than the sounds of the right side. Then, owing to these facts, endocarditis could exist to the same extent in the right side as in the left, and yet escape our observation.

Admitting that endocarditis is most generally found in the left side of the heart alone, why is this the case? This question brings us to the causes of endocarditis before it can be answered, and before considering the special causes we must notice the anatomical structure of the endocardium.

The endocardium is a perfect serous membrane, while the pericardium is a fibro-serous membrane. All the valves of the heart are but re-duplications of this same endocardial membrane, which serve as guards or sentinels at every portal of every chamber of the heart, to prevent regurgitation or the backward movement of the blood during the systolic action of the organ.

Of the different idiopathic causes of endocarditis, acute inflammatory rheumatism and Bright's disease of the kidney stand pre-eminent.

I have never regarded endocarditis existing with, or resulting from, Bright's disease as an active inflammation —such as is found with inflammatory rheumatism—but rather a low degree of passive inflammation, the result of an impoverished blood and a loss of nutrition. It is a well know fact that when the blood is drained of its rich nutrient properties such as albumen, fibrin, etc., (as is the case in Bright's disease) the different organs and tissues of the body must perish by a slow and low degree of inflammation, ending in suppuration; and such is the case in endocarditis from Bright's disease. The endocardium being a serous membrane, is sensitive and highly vascular, and having an unending and laborious duty to perform, necessarily requires vast nutrition from a healthy blood; hence we can easily understand why it undergoes a low degree of inflammation, and finally softening, as the result of albuminuria.

This I believe to be the case of this affection, when in connection with Bright's disease.

Endocarditis results from acute inflammatory rheumatism much more often than from Bright's disease; indeed more often than from all other idiopathic sources. Every competent physician knows with what care and anxiety he examines and scrutinizes the condition of the heart during every visit to his patient afflicted with acute rheumatism, and in fully one-half of his patients he will find endocarditis existing during the highest inflammatory excitement. Sometimes it does not make its appearance until the favorable decline of the rheumatic inflammation, when it can then be said to exist as a consecutive.

It has never been definitely decided as to why endocarditis should result from inflammatory rheumatism; but most all agree that it is the influence of lactic acid on the membrane. It is an established fact that in acute articular rheumatism there is a superabundance of lactic acid, and certainly it is due to this poisonous agent that the endocardial membrane suffers.

It is not our intention, in this article, to discuss the source or cause of lactic acid in the system; suffice it to say that it is the result of chemical and fermentative changes in the system. It may be produced in the digestive system, in the blood itself, or in the tissues themselves.

Lactic acid must not be mistaken for uric acid. Uric acid is one of the natural constituents of the urine, and may be increased to an alarming extent by the chemical decomposition of certain alkaline agents taken into the system.

We come now to the cause of endocarditis in the left side of the heart when found there alone. No doubt exists as to its being caused by lactic acid poisoning.

The question naturally arises as to how and why this poison affects the left endocardium all the time, and the right only occasionally. The answer is that this morbid acid comes directly in contact with the left and not with the right endocardium; and right here springs a nice point. Most writers claim that the acid is generated in the lungs during the passage of the blood to and fro, with the action of the atmosphere upon it, and that this newly generated poison comes directly in contact with the left or arterial heart, and spends its force before making the circuit of the circulation. This may be true, but we have never been able to agree fully with the statement. We agree that the poison does come directly in contact with the left endocardium, and that it exerts its poisonous effects there ; but we do not agree that it is generated in the lungs during the passage of the blood with the action of the atmosphere upon it; but that it is generated in the blood and tissues of the body through the process of chemical decomposition, and all this takes place long before the blood returns even to the right side of the heart. Another question presents itself thus : Why does it not affect the right endocardium then as well and to the same extent as the left endocardium ?

My own individual answer is, that the acid being generated in the system returns to the right side of the heart in the venous blood, in company with a large amount of carbonic acid; now it is well known that carbon and carbonic acid are agents that prevent the decomposition of organic substances to a great extent; also that they will prevent poisonous acids or gases from exerting their deleterious effects upon the different tissues of the body. Now this poisonous acid is held in subjection by the carbonic acid as long as the carbonic acid is allowed to remain in the blood with it; but when both with the blood pass through th[illegible]ght or venous heart into the lungs, there the carbonic acid [illegible] expelled by exhalation, while the lactic acid remains [illegible]nopposed, and, of course, attacks the left side of the heart long before it again comes in contact with the carbonic acid. This theory seems to me (while it is strictly my own) far more plausible than that the lactic acid is generated in the lungs themselves.

Endocarditis from lactid acid poisoning presents us a different pathology from that found from any other cause. We not only have the thickened, turgid membrane, with thickened valves, but we have roughened edges of valves and warty vegetations of growths from the edges of the valves, and the edges of the different chambers of the heart.

I do not believe that we would have the extremely thickened condition of both valve and lining membrane, with warty growths and contracted orifices in any other form of endocarditis than that of lactic acid poisoning; but as we are not to dwell on the pathology of this disease, we will now pass to its diagnosis, and to its discrimination from pericarditis.

Endocarditis, like all other diseases, does not present all the symptcms attributed to it in every case. Indeed, in many ordinary cases, some of the symptoms will be wanting, even the bellows murmur, or blowing sound, as it is commonly called. It is not always easily diagnosed, and we are often left in doubt as to whether it is a case of endocarditis or some other affection giving rise to the same obscure symptoms.

In a true case of endocarditis, with valvular involvment, we cannot be readily mistaken as to its certainty, particularly if it accompanies acute rheumatism. As we stated in the beginning, endocarditis can be either extensive or circumscribed. It can involve a large portion of the lining membrane, including the valves, or only one valve or a small patch of membrane most distant from any valve.

Circumscribed endocarditis, without valvular implication, will not further claim our attention, as all the symptoms by which we are enabled to arrive at a co[illegible]t diagnosis, belong to the disease when developed wi[illegible]al-vular inflammation, with or without deposits.

Before speaking of the special symptoms [illegible]his inflammation we will say that every symptom belonging to this disease will be found singly or collectively in many other functional or organic affections of the heart. And it is only after we get the history of the case, or find the most of these symptoms in connection with rheumatism or Bright's disease, that we can fully determine it to be a case of endocarditis.

To group the symptoms and physical conditions will engage our attention now. In a well developed case of endocarditis we have more or less pain in the interior of the heart; increased action of the heart with augmented force against the chest; pulse quicker and stronger than natural, particularly during the acute inflammatory stage; sometimes irregular action of the heart, owing to valvular obstruction. We most always have valvular obstruction when we have thickened valves from inflammatory deposit on the surface or warty vegetation on their edges; or, further, by lymphatic deposit on the edges of the orifices themselves.

But one of the most prominent symptoms of all is the blowing sound, or belows murmur, heard in the region of some valve or valves. It is heard most often over the mitral and left semi-lunar valves; but the mitral valve alone is the one most often affected.

Why this murmur? Our authors nearly all agree that it is due to the roughened edges of the valves from fibrinous deposit and warty growths. This is true, but I believe the cause goes further than this. It is due also to the diminished calibre of the orifices, in consequence of this same lymphatic deposit on their edges. We often find the patient with a dry, hacking cough, difficulty of breathing and unable to lie down. Nearly all, and sometimes all, these symptoms and physical conditions are found in well developed cases of endocarditis.

No percussion dullness exists as is the case in pericarditis; no prominence of chest or intercostal spaces in the cardial region; no suppressed action of heart; no friction sound, as is the case in pericarditis.

In pericarditis, before effusion takes place, we have an audible friction sound in the præcordial region; later a suppressed distant sound, owing to effusion in the pericardial sac. Heart at first excited and strong with strong impulse; later, action weak with feeble circulation. Pain at first near the surface of the heart and in the pericardium; later, little or no pain at all. Percussion dullness often increases in extent as the pericardium becomes distended with fluid. These are the symptoms and physical conditions of pericarditis as contrasted with those of endocarditis. A few words about the symptoms and physical signs of endocarditis and we are done.

We must not believe that any or all of these symptoms belong exclusively to endocarditis, certainly not.

The bellows murmur, one of the most important of all symptoms, is heard in other conditions of the heart. It will be heard in rupture of the mitral or aortic valves, in ossification of valves in old age, in the aortic orifice with mitral obstruction, causing the left ventrical to contract upon a small current of blood, forcing it through the aortic orifice—and who has not heard this same blowing sound in cases of protracted anæmia from malarial poisoning, chlorosis and other blood dyscrasia?

Pain in the interior of the heart can be due to ruptured valves, carditis proper, neuralgia and many other causes. Rapid action of heart, with strong impulse, can be due to all other acute inflammations of the heart, lungs or pleura. Cough can be due to bronchitis, pneumonia, pleurisy, pericarditis and many other affections of the thoracic organs. Difficulty of breathing exists in all these affections. So we see that we cannot rely strictly upon any special symptom without first finding a factor for the suspected disease, then grouping together all the symptoms and making a most thorough physical examination, we may be able to diagnose a case of true endocarditis.

